# Electro-Mechanical Properties of Multilayer Graphene-Based Polymeric Composite Obtained through a Capillary Rise Method

**DOI:** 10.3390/s16111780

**Published:** 2016-10-25

**Authors:** Chiara Acquarelli, Licia Paliotta, Alessio Tamburrano, Giovanni De Bellis, Maria Sabrina Sarto

**Affiliations:** 1 Department of Astronautical, Electrical and Energy Engineering of Sapienza University of Rome (DIAEE), Via Eudossiana 18, Rome 00185, Italy; licia.paliotta@uniroma1.it (L.P.); alessio.tamburrano@uniroma1.it (A.T.); giovanni.debellis@uniroma1.it (G.D.B.); mariasabrina.sarto@uniroma1.it (M.S.S.); 2 Research Center for Nanotechnology Applied to Engineering of Sapienza University (CNIS), Rome 00185, Italy

**Keywords:** strain sensor, polymer composite, graphene nanoplatelets, capillary rise, piezoresistivity

## Abstract

A new sensor made of a vinyl-ester polymer composite filled with multilayer graphene nanoplatelets (MLG) is produced through an innovative capillary rise method for application in strain sensing and structural health monitoring. The new sensor is characterized by high stability of the piezoresistive response under quasi-static consecutive loading/unloading cycles and monotonic tests. This is due to the peculiarity of the fabrication process that ensures a smooth and clean surface of the sensor, without the presence of filler agglomerates acting as micro- or macro-sized defects in the composite.

## 1. Introduction

Structural health monitoring (SHM) has emerged as an effective technique to monitor the integrity of engineered structures in both civil and aeronautical fields [1,2]. It typically employs sensors attached to or embedded into the structures and networked, to provide real-time surveillance of structures and equipment. These sensors rely on different physical properties, such as optical, piezoelectric and piezoresistive effects [3,4,5]. Recently, piezoresistive strain gauges based on polymer matrix filled with carbon nanostructures, such as carbon nanotubes (CNT) [6,7], reduced graphene oxide (rGO) [8,9], graphene nanoplatelets (GNP) or multilayer graphene nanoplatelets (MLG) [10,11,12,13,14,15], have gained considerable attention from both academia and industry due to their high sensitivity, mechanical compatibility with the host structures, isotropic response and size scalability. These types of sensor are typically made of polymer composites filled with carbon nanostructures, which create a percolating electrical network, whose resistance is dependent on the distance between particles and on the piezoresistivity of the particles themselves [16]. Recently, a few studies have been performed in order to assess how the electromagnetic properties at radiofrequency and in the microwave of polymer composites filled with carbon-based nanostructures vary as a function of the composite elongation [17,18].

The polymer matrix in a composite has the function of transferring applied loads to the reinforcing fillers and to provide inter-laminar shear strength. The filler-to-matrix interface governs the load transfer mechanism, and for strain sensor applications, it influences the obtainment of a stable piezoresistive characteristic, over subsequent measurements, as well as in case of cyclic loading [19,20]. Nevertheless, process-induced defects in the composite, such as voids and filler agglomerates, may originate microcracks [21,22], which are enhanced by induced residual stresses and distortions caused by manufacturing and machining processes [23,24]. These defects result in a progressive deterioration of the mechanical properties of the composite for increasing or repetitive load cycles, and they limit the use of such composites in strain sensor applications because of the continuous increase of the electrical resistance of the material under stress [13,25,26]. The investigation of the role that the composite microstructure plays on the cyclic piezoresistive response and on the hysteretic behavior of strain sensors has attracted considerable research efforts [9,10,13,19,27]. Recently Zha et al. have produced a strain sensor based on functionalized GNP/epoxy composite for in situ damage monitoring of structural composites by a resin casting method [10]. The composite sensor has shown a relatively good sensitivity, with a gauge factor (*GF*) of ~45 and a Young's modulus of ~2.2 GPa. However, during tensile loading, the normalized electrical resistance variation of the composite sensor increases linearly at the beginning, while subsequently showing a nonlinear drift and an irregular ladder-shaped growth corresponding to microcrack accumulation and permanent microstructure damage of the sensor. Moreover, Tung et al. have developed a piezoresistive sensor made of rGO/epoxy composite at 2 wt %, with a *GF* of 12.8. Surface functionalization of the filler enabled the improvement of the filler-matrix interface, which is critical for the sensing performance of the rGO/epoxy composite [9]. The reversibility and the damage detection capability of the sensor were monitored as a function of increasing mechanical strain through measurements of the resistance variation under cyclic loading. When the strain exceeded the value of the elastic domain boundary of the epoxy matrix, the resistance started deviating from the linear curve probably due to the degradation of the graphene-based composite caused by microcracks at the matrix-filler interface [9]. In a previous study, we proposed a new method to estimate the average size and dimensions of GNP agglomerates in epoxy-based composites, and we demonstrated the degradation of the mechanical properties of the composite due to the presence of filler agglomerates through dynamic thermo-mechanical analysis [28].

In the present study, we propose a novel method to produce thin laminae made of MLG-filled vinyl-ester composites featuring high stability of the electromechanical response for the application as strain sensors. A MLG-composite lamina is obtained via spontaneous capillary-driven filling (SCDF) of microchannels with an MLG-polymer mixture at 1 wt % of MLG. The SCDF of microchannels has been widely studied for different applications, such as ink-jet printing, lab-on-chip and underfilling of a flip chip [29,30,31,32], but to the best of our knowledge, it has never been applied to produce a strain sensor with a highly reliable response. The aim of this work is to take advantage of the SCDF method in order to achieve maximum stability of the sensor response under loading/unloading conditions and monotonic increasing load. 

The thin composite was produced using a solution processing technique. Rheological characterizations of both the plain polymer and of the MLG-polymer mixture were carried out using a rotational rheometer operating in steady shear state mode. The capillary rise of the colloid in vertical channels was recorded with the aid of a CCD camera (Nikon D7100 equipped with Tamron 90 mm F2.8 macro lens, Nikon, Tokyo, Japan) in order to measure the meniscus motion during the capillary rise. The microstructure of the produced samples was investigated through field-emission scanning electron microscopy (FE-SEM). The electrical, mechanical and electromechanical characteristics of the MLG-composite lamina have been assessed experimentally. The efficiency of the proposed SCDF method to produce thin composite lamina for strain sensing was finally validated through the comparison with a sensor produced by cast molding and sawing. 

## 2. Materials and Methods

**Figure 1:**
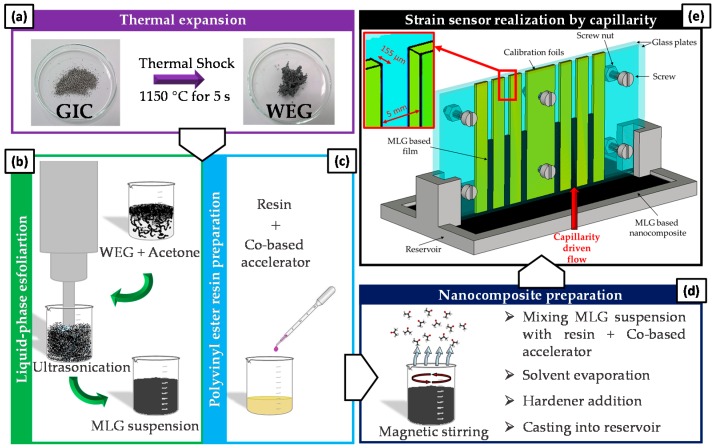
Schematic of the MLG/resin composite laminae fabrication route. GIC, graphite intercalation compounds; WEG, worm-like expanded graphite. (**a**) photographs of GIC as purchased and WEG obtained after thermal shock; (**b**) schematic of the MLG suspension preparation; (**c**) schematic of the composite solution processing technique; (**d**) nanocomposite preparation steps; (**e**) capillary rise setup scheme.

MLG/resin composite laminae are produced starting from an MLG/resin mixture, via spontaneous capillary-driven filling of a rectangular microchannel. The process is sketched in Figure 1 and described in the following.

### 2.1. MLG Suspension Preparation

MLGs are produced by liquid phase exfoliation of thermally-expanded graphite intercalation compounds (GIC) provided by Graftech Inc. (Parma, OH, USA) as described elsewhere [28]. The intercalated precursor is expanded at 1150 °C for 5 s in a muffle furnace, forming a worm-like expanded graphite (WEG), which is dispersed in acetone. The obtained suspension is sonicated using an ultrasonic probe (Vibracell VC 505, Sonics & Materials Inc., Newtown, CT, USA) operating at 20 kHz with an amplitude of 70% for 20 min, set in pulse mode (1 s on and 1 s off), at the constant temperature of 15 °C. The probe tip was immersed at a fixed depth from the suspension free surface of ∼2.5 cm. 

### 2.2. MLG/Polyvinyl Ester Resin Composite Laminae Realization 

The setup used for the production of the composite lamina through the SCDF method is sketched in Figure 1e. The capillary channels have a length of 80 mm, a width of 5 mm and thickness *δ*, which is set to 155 µm through the use of calibration foils positioned between two glass substrates tightened with screws.

The resin used in all of the experiments is a commercially available epoxy-based vinyl ester product (DION 9102, Reichhold, Durham, NC, USA). The liquid resin has an initial viscosity of 150–200 mPa·s, a density of 1.01–1.05 g/cm^3^ and a styrene content around 50 wt %. The composite is produced using a solution processing technique. First, the liquid vinyl ester resin is mixed with 0.2 wt % of a Co-based accelerator (Accelerator NL-51P, Akzo Nobel Polymer Chemistry, Arnhem, The Netherlands) (Figure 1c). The resin mixture is then poured into a beaker containing the MLG suspension, previously obtained via ultrasonication (Figure 1b). The prepared MLG/resin mixture is further sonicated for 30 s using an amplitude of 40%, with the aim of improving the dispersion of the filler into the polymer mixture. The solution is then magnetically stirred at 200–250 rpm to remove the solvent in excess (Figure 1d). Upon complete removal of the solvent, the hardener is added at 2 wt % (Butanox LPT, Akzo Nobel Polymer Chemistry, Arnhem, The Netherlands). The resulting MLG/vinyl ester mixture (MLG/resin mixture plus hardener) is stirred at 250 rpm for a few minutes and finally cast in the reservoir of the capillary rise setup (Figure 1e). The resulting composite is cured in air for 24 h and post-cured for 24 h in an oven at 70 °C. 

In order to assess the influence of the lamina manufacturing process on the electromechanical response of the sensor, the MLG/vinyl ester mixture is also poured in a mold with dimensions of 16 mm × 8 mm × 6 mm and cured following the same procedure described above. The obtained brick-shaped sample is successively cut (in the central position) using a Buehler^®^ IsoMet 4000 precision saw, in order to obtain a composite lamina with the same thickness of the one obtained through the SDCF method. 

### 2.3. Rheological Characterizations

Rheological characterization of both the plain resin and the MLG/resin mixture is carried out using a rotational rheometer (MCR302, Anton Paar, Graz, Austria), operating in steady shear state mode. The measurements are performed at 23 °C using a Peltier-controlled temperature hood, employing a 50-mm plate-plate geometry. Apparent viscosity is measured in the range of shear rates from 0.1 s^−1^ to 100 s^−1^, with a gap between the plates of 0.7 mm–0.8 mm.

### 2.4. Measurement of the Rise Height during SDCF

The height *H* of the flow front of plain resin and of the MLG/resin mixture (both with and without hardener) is recorded with the aid of a CCD camera during the capillary rise. 

### 2.5. Morphological Characterizations

The morphology of the top surface and of the cross-section of the produced samples is investigated by scanning electron microscopy (SEM) using a Zeiss Auriga Field Emission-SEM (FE-SEM, Carl Zeiss, Oberkochen, Germany) available at Sapienza Nanotechnology and Nanoscience Laboratory (SNN–Lab). For cross-section analysis, the samples are fractured in liquid nitrogen, and a 10-nm Cr film is sputtered on the fracture surfaces using a sputter coater (Q150T, Quorum Technologies Ltd., Laughton, UK). 

### 2.6. Electromechanical Characterization of Strain Sensors

**Figure 2:**
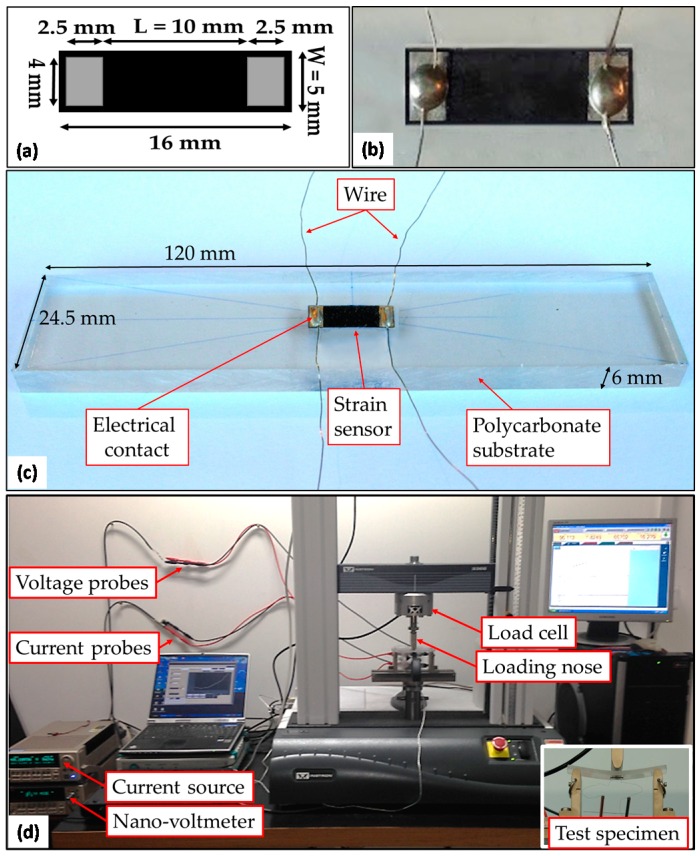
(**a**) Dimensions of the strain sensors with electrical contact areas; (**b**) photograph of the realized sensor; (**c**) photograph of a test bed, including a strain sensor attached over a polycarbonate beam; (**d**) electromechanical test setup, including the test bed, voltage probes, source meter, nanovoltmeter and laptop computer.

Strain sensors are fabricated using MLG-composite laminae produced either through SDCF or sawing the brick-shaped specimen. For this purpose, the composite laminae of thickness t are cut in rectangular samples having dimensions of 5 mm × 16 mm. Electrical contacts are realized at both extremities as sketched in Figure 2a. At first, a thin silver-paint layer (Electrolube^®^) is deposited on rectangular areas of 4 mm × 2.5 mm. Then, after silver-paint drying, a silver-based epoxy adhesive (CircuitWorks^®^) is applied over the contacted areas to attach tin-coated copper wires (0.2 mm in diameter). Finally, the sensors are cured in an oven at 70 °C for 15 min to promote polymerization of the silver-based epoxy adhesive. A photograph of a sensor is shown in Figure 2b.

The electromechanical characterization of the produced sensors is performed in flexural mode through a three-point bending test. For this purpose, the strain sensor is bonded over a polycarbonate beam 6 mm in thickness, 120 mm in length and 24.5 mm in width, using a cyanoacrylate-based adhesive (Figure 2c). 

Before testing, all test beds are stored in a desiccator for 48 h. The electromechanical tests are carried out in a controlled environment, i.e., at 23 ± 0.5 °C and 40% ± 5% relative humidity.

At first, the initial DC electrical resistance $R_{0}$ of the sensor is measured applying the two-wire volt-amperometric method. The test is performed in delta mode using a Keithley 6221 DC/AC current source connected to a Keithley 2182a nano-voltmeter (Keithley Instruments, Cleveland, OH, USA), remotely controlled by a PC for data acquisition and analysis. 

The thickness t of each lamina is measured using a digital micrometer (Mitutoyo, Takatsu-ku, Kawasaki, Japan) and it is estimated as the average of six measurements performed on a grid of six different points over the sample. 

Next, the electromechanical response of the strain sensor is obtained measuring the variation of the DC electrical resistance as a function of the applied flexural strain during the three-point bending test, according to the American Society for Testing and Materials (ASTM) D 790. The flexural tests were performed with a span-to-depth ratio of 16:1, as suggested by the standard. The experimental setup is shown in Figure 2d. 

**Figure 3:**
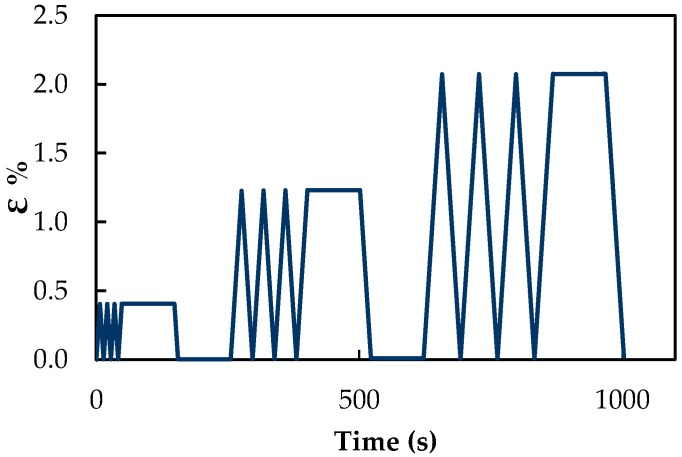
Applied strain as a function of time during one loading/unloading cycle.

Two different tests are carried out on each sensor. At first, six loading/unloading cycles (in the following indicated as “cyclic test”) are applied to the test bed. Each cycle consists of three triangular/trapezoidal loading/unloading profiles with incremental maximum strain levels, i.e., 0.4%, 1.2% and 2.1%, as shown in Figure 3. The tests are performed under displacement control with crosshead speed set at 10 mm/min. Secondly, a series of five consecutive tests, each one consisting of a monotonically-increasing load (in the following, indicated as “monotonic test”) is applied with a crosshead speed set at 1 mm/min, up to a maximum strain of 1.5%. It is worth noting that in both test typologies the maximum applied strain is within the elastic range of the polycarbonate substrate (i.e., 0–390 N, which corresponds to 0–63 MPa). 

The initial resistance of the sensor (i.e., without an applied strain) measured before the loading/unloading cycles is indicated as $R_{0}$. The initial resistance of the sensor measured at the beginning of the second type of tests is indicated as $R_{0s}$. The engineering gauge factor (*GF*) of the sensor is defined as:

(1) $$GF=\;\frac{\Delta R}{R_{0}\varepsilon }$$

where $\Delta R=R\left(\varepsilon \right)-R_{0}$ is the electrical resistance variation and ε is the flexural strain.

## 3. Results and Discussions

**Figure 4:**
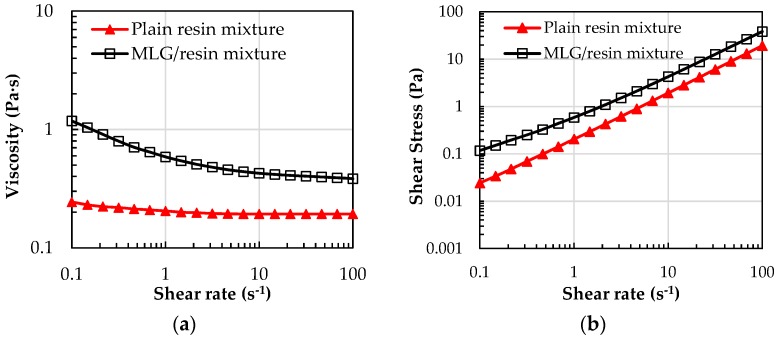
(**a**) viscosity and (**b**) flow curves of the plain resin and of the MLG/resin mixture.

Figure 4a,b shows viscosity and flow curves of the plain resin and of the MLG/resin mixture, measured at 23 °C. The viscosity measured for the neat resin falls within the 190 mPa·s–240 mPa·s range, in perfect agreement with data declared by the manufacturer. While the plain resin shows a Newtonian behavior, with a small deviation from ideality only for the low shear rate regime (<3 s^−1^), the MLG/resin mixture features a shear thinning tendency for low shear rates, leading to a Newtonian-like plateau only for high shear rates: this trend is associated with the orientation of the plate-like filler along the flow direction, as already stressed by Kim et al. [33]. Moreover, the addition of MLGs to the resin leads to an almost five-fold increase in the initial viscosity (for the lowest shear rate), rising from 244 mPa·s for the plain polymer to 1180 mPa·s for the 1 wt % MLG/resin mixture, thus indicating a strong particle-particle interaction, as already reported by other groups for vinyl ester resins filled with carbon-based nanomaterials [34].

Figure 4b highlights also the development of an apparent yield stress for the mixture (i.e., the minimum shear stress that has to be applied to the liquid mixture to let the flow begin), when MLGs are added to the polymer, suggesting the formation of a filler-interconnected network for low shear rates [35].

**Figure 5:**
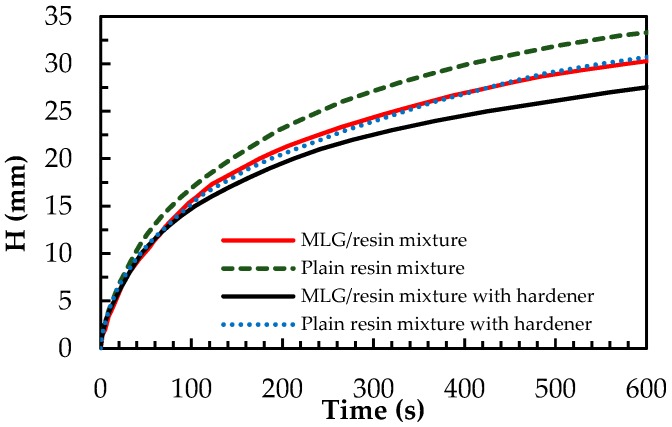
Variation of rise height *H* as a function of time of plain resin and of the MLG/resin mixture, with and without hardener.

The results of the rheology characterizations are consistent with the observed rise height H inside the capillary channels, of the plain resin and of the MLG/resin mixture, as a function of time (Figure 5). It is observed that in all cases, H increases quickly at the initial stage of the process, owing to relatively small resistances from gravity and viscous forces in the fluid volume. The resistance from gravity increases significantly with further liquid rise, while the driving capillary force (defined as the pressure difference between the inlet of microchannel and the flow front) remains relatively constant. Consequently, H approaches asymptotically an equilibrium value (i.e., the maximum rise height $H_{\max}$), corresponding to the balance between capillary and gravity forces [31]. The addition of MLG to the resin, even at the low concentration of 1 wt %, produces an increase of the viscosity of the mixture, and consequently, the capillary rise of the composite mixture becomes slower [32]. A similar trend is observed in the presence of the hardener, which modifies the viscosity of the mixture. Table 1 shows the maximum rise heights observed with the different systems. 

### 3.1. Morphology

**Figure 6:**
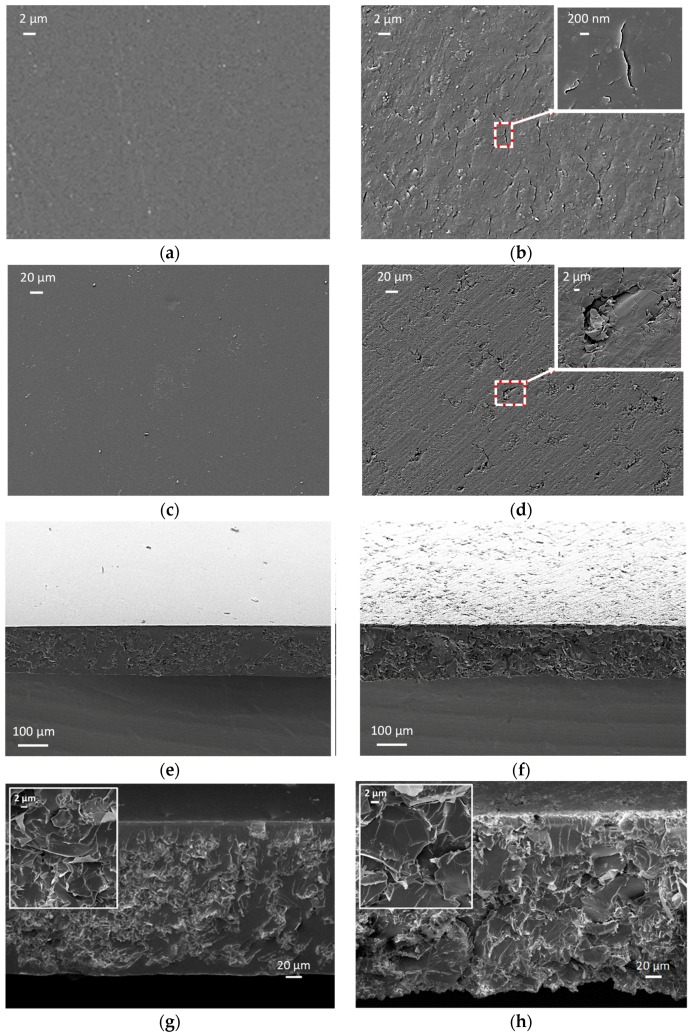
Top views of: vinyl ester lamina produced via (**a**) the capillary rise method or (**b**) the resin casting method; MLG-composite lamina produced via (**c**) the capillary method or (**d**) the resin casting method. Cross-section views of: MLG-composite lamina obtained via (**e**) the capillary method or (**f**) the resin casting method. High magnification and zoomed areas of the cross-section of MLG-composite lamina obtained via (**g**) the capillary method or (**h**) the resin casting method.

The microstructure of laminae made of plain resin or MLG/resin composite, fabricated either through capillary rise or resin casting in a mold and subsequent sawing of the composite sample, is investigated by SEM, as shown in Figure 6a–h. 

Figure 6a,b shows the top views of plain resin laminae, obtained via capillary rise and casting, respectively. Figure 6a clearly shows the surface smoothness of samples obtained through capillary rise. On the contrary, the presence of microcracks and voids in the second type of samples (produced by sawing of the brick-shaped specimen) are the result of residual stresses induced during the curing step in the three-dimensional mold and of the subsequent manufacturing process. These defects have an average size of 200 nm–2 µm. As a comparison, Figure 6c,d shows the top views of MLG-composite laminae produced through the two methods. The surface of the lamina produced through capillary rise is smooth and does not show relevant imperfections nor MLG agglomerates emerging over the sensor surface, nor micro-/meso-scopic defects. On the contrary, the sample obtained through the latter method is characterized by flaws and microcracks, which are representative of the presence of large filler agglomerates, having average dimension of 2–20 µm, and appear over the exposed surface of the sample during the sawing process. 

Next, the cross-sections of the MLG-composite laminae prepared through the two different methods are analyzed. Figure 6e shows that the samples produced through capillarity rise have an outer shell of resin, having a thickness up to ~10 µm, with a very low filler loading and without agglomerates, whereas a higher filler concentration and the presence of agglomerates are observed underneath the surface of the sample. Furthermore, SEM micrographs show evidence of a thorough grafting of the polymer onto the MLGs with a good integration between filler and matrix, probably favored by shear forces acting on the mixture during the capillary process. On the other hand, in samples obtained via the resin casting method, MLGs and their agglomerates are exposed over the sample surface (Figure 6f). Figure 6g,h shows a higher magnification of the previous micrographs and the details of filler agglomerates. From the zoomed area, it can be appreciated that MLGs are folded and stacked within the sample cross-section as a result of the fabrication process (capillary rise), which induces an orientation of the fillers and of their agglomerates along the flow lines. On the contrary, filler and filler agglomerates are randomly distributed within the sample produced by resin casting, so that MLGs do not show a preferred orientation (as observed in the detail of Figure 6f), resulting in a more uniform distribution of the filler across the whole sample section, as compared to the capillary rise-produced samples.

### 3.2. Electrical and Electromechanical Characterization of MLG-Composite Sensors

At first, the DC electrical resistance $R_{0}$ and thickness t of the MLG-composite sensors produced through capillary rise are measured. It results in $R_{0}$ = 57 ± 14 kΩ and t= 160 ± 5 µm. The obtained values are in line with the ones reported in our previous studies [16,36,37]. 

**Figure 7:**
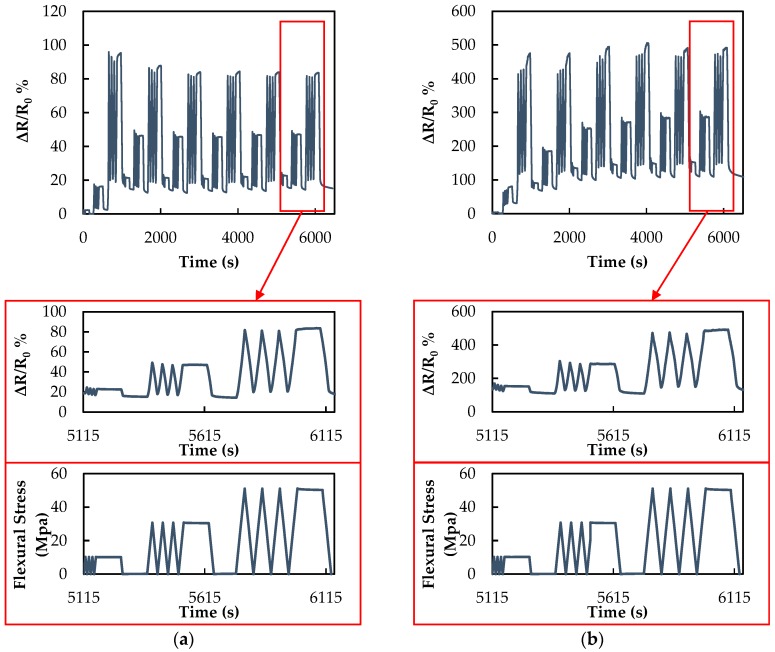
(**a**) normalized variation of electrical resistance $\Delta R/R_{0}\%$ and flexural stress during strain cycling for a sensor produced via the capillary rise method; (**b**) normalized variation of electrical resistance $\Delta R/R_{0}\%$ and flexural stress during strain cycling for the sensor produced via the resin casting method.

Next, the electromechanical response of the fabricated MLG/resin composite sensors is analyzed. Figure 7a,b shows the variation over time of the normalized electrical resistance, expressed as percentage ($\Delta R/R_{0}$%), during quasi-static loading/unloading cycles, for strain sensors produced either via capillary rise or resin casting in a mold. These tests are conducted in order to investigate the sensitivity and reliability of the sensor response. The graphs clearly show that $\Delta R/R_{0}$% of both sensors accurately follows the applied strain profiles for increasing strain levels.

It should be noted that $\Delta R/R_{0}\%$ of the sensor produced via capillary rise, after the first cycle, shows a drift of 18% at rest. This value remains almost constant within a variation of 1% during all of the successive cycles when ε = 0. For the same sensor, the value of $\Delta R/R_{0}\%$ measured when the strain is kept constant for 100 s (plateau of the trapezoidal profile of the stress-strain curve) stabilizes at ~82% after the second cycle, until the end of the test. This suggests that the piezoresistive properties of the composite have not been subjected to degradation after a total of 72 quasi-static loading/unloading ramps.

On the contrary, the sensor produced through resin casting is characterized by a progressively-increasing value of $\Delta R/R_{0}$% when ε = 0. It stabilizes around 110% only after five cycles. It is also observed that $\Delta R/R_{0}$% measured during the strain plateaus is subjected to an increase of more than 10% of its initial value and does not stabilize even after six consecutive cycles (corresponding to a total of 72 quasi-static loading/unloading ramps). This result can be attributed to the deterioration of the piezoresistive properties occurring when the sensor, subjected to cycles at increasing strain levels, changes its microstructure because of the stress localization around the filler agglomerates, acting as surface defects, resulting from the manufacturing process.

The deterioration of the piezoresistive properties of polymer composites filled with carbon nanostructures during cyclic mechanical tests with incremental strain level has been already observed in [10,38,39]. In particular Zha et al. [10] report that during incremental cyclic tensile loadings, even after the third cycle, $\Delta R/R_{0}$% does not return to its initial value, demonstrating the unstable drift of the piezoresistive response. Actually, we assume that in this type of sensor, incremental strain induces primarily a partially-irreversible change of the morphology of the agglomerates located over the lamina surface, in which nanostructures are kept together by weak forces, with a consequent increase of the distance between nanoparticles, resulting in the break-up of conductive networks. Actually, in [40], the resistance drift at rest was attributed to the cracks generated in the smooth pencil-trace film. Moreover, in [41], it is reported that in a sensor made of CNT-filled composite, $R/R_{0}$ has a drift during subsequent cycles, which can be explained with the rearrangements of the CNT network due to the viscoelastic nature of the matrix; in addition, the fact is noted that $R/R_{0}$ does not recover to its initial value, which can be attributed to the permanent modification of the morphology of the CNT network.

Bautista-Quijano et al. obtained a similar behavior during mechanical loading–unloading cycles. They reported that the piezoresistive signal of the nanocomposite strain sensor did not return to the initial value after the end of each cycle due to hysteresis. This phenomenon could be related to the rearrangement of the nanoparticles after the first mechanical loading [38]. Moreover, Vertuccio et al. [39] pointed out that the resistive behavior of multi-walled carbon nanotube (MWCNT)/epoxy resins, tested under mechanical cycles at increasing levels of strain, present microscale damages that are revealed by the presence of a residual resistance, which increases with the amount of strain accumulated in the matrix.

We also notice from Figure 7a,b that the sensor is characterized by a slightly non-symmetrical response when subjected to loading and unloading symmetrical ramps. This phenomenon was attributed in [41] to the presence of bumps, occurring in return steps, originated by fiber buckling. In our case, the asymmetric response of the sensor can be ascribed to a combination of its nonlinear piezoresistive characteristic (see monotonic test below) and of the viscoelastic behavior of the polymeric matrix and polycarbonate substrate [42].

The magnified views of the last cycle of the electromechanical responses reported in Figure 7a,b show the normalized variation of electrical resistance $\Delta R/R_{0}\%$ of the sensor and the flexural stress applied to the polycarbonate substrate. The reported results show that when the response of both sensor types is stabilized, during the 100 s in which the applied stress is kept constant, $\Delta R/R_{0}\%$ exhibits slight variations, which can be attributed to the viscoelastic nature of the polymer. The outer polymer shell surrounding the composite lamina produced through capillarity rise, appearing in Figure 6c, has the role of preventing MLG agglomerates from emerging over the sensor surface and to behave as defects. As a consequence, the electromechanical response of the sensor produced through capillarity rise is characterized by a better repeatability and higher stability than the sensor produced through resin casting in a mold.

**Figure 8:**
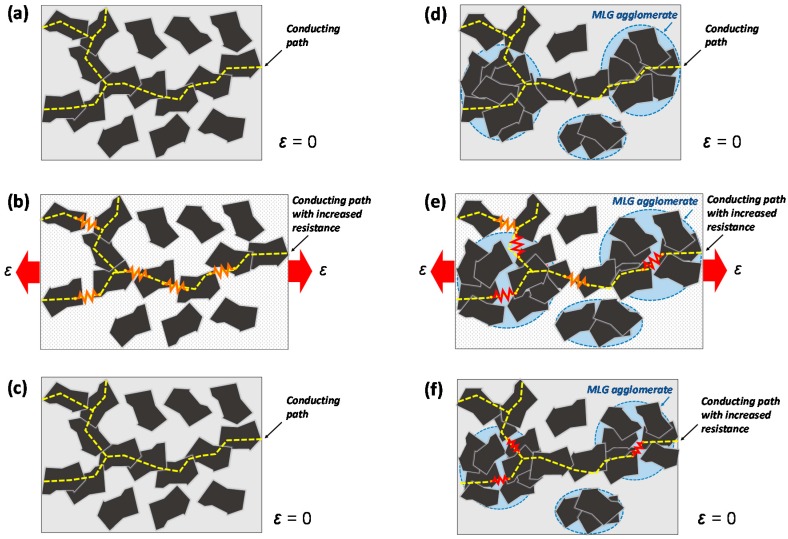
Schematic representation of the role of MLG agglomerates on the conductive network changes in a composite, subjected to loading and unloading: composite in which the presence of MLG agglomerates is negligible (**a**–**c**) or not (**d**–**f**).

The role that agglomerates play in a composite as regards its electrical conducting properties, when the composite itself is subjected to a mechanical stress, is explained using the sketch of Figure 8. The drawings show different conducting mechanisms occurring in an MLG-filled composite when the presence of filler agglomerates is negligible (Figure 8a–c) or it is relevant (Figure 8d–f). In the former case, MLG agglomerates are not present. Therefore, the applied stress distributes uniformly within the composite, thus guaranteeing a high reversibility of the sensor response under a cyclic mechanical solicitation. This mechanism can be considered to be the dominant one in the sensor produced through capillarity rise, because the presence of MLG agglomerates in the outer polymer shell (shown in Figure 6g) is negligible. On the contrary, in the second case (Figure 8d–f), the conducting mechanism under an applied stress in a composite in which the presence of MLG agglomerates is not negligible is sketched. Agglomerates behave as defects that induce a localization of the applied stress and a consequent non-fully-reversible electromechanical response of the sensor. Actually, when the strain is released, agglomerates do not restore their original morphology due to the weak interactions taking place among nanostructures: this results in an increase of the overall resistance of the conductive networks. Such a mechanism can be considered to be the dominant one in the sensors produced through sawing of the bulk composite, in which the presence of MLG agglomerates over the surface and inside is not negligible, as shown in Figure 6b,h.

**Figure 9:**
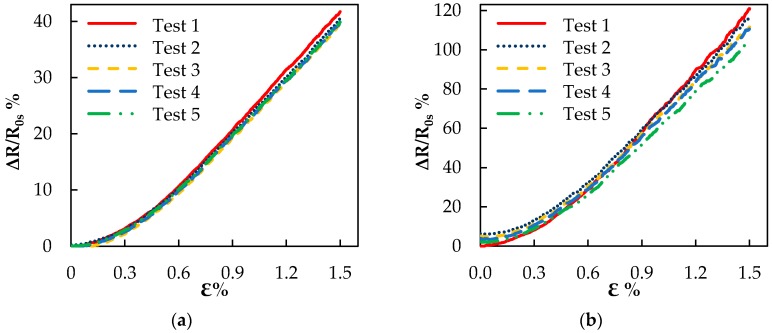
(**a**) Normalized variation of electrical resistance $\Delta R/R_{0s}$% as a function of strain during monotonic tests for sensor produced via the capillary rise method; (**b**) normalized variation of electrical resistance $\Delta R/R_{0s}$% as a function of strain during monotonic tests for the sensor produced via the resin casting method.

Finally, Figure 9a,b shows the piezoresistive response of the two sensor types during the monotonic test. The variation of the normalized electrical resistance $\Delta R/R_{0s}\%$ for the sensor produced via capillary rise shows approximately a linear trend above ~0.3% strain. Such linearity is maintained all through the consecutive tests, without relevant drifting. In particular, it is observed that after each test, when the applied strain returns to zero, the resistance assumes its initial value, as a proof of the sensor integrity. The maximum final value of $\Delta R/R_{0s}$% (at 1.5% of strain) is 40%, already after the second test. 

In the case of the sensor produced through resin casting in a mold, the linearity of the piezoresistive response above ~0.3% of the strain is maintained, but $\Delta R/R_{0s}\%$ does not return to zero after the end of each test. Furthermore, the maximum final value of $\Delta R/R_{0s}$% at 1.5% of the strain decreases from ~120% at the first test to ~100% at the fifth one.

From the curves of Figure 9a,b, it is also possible to determine the gauge factor (*GF*) of the sensor. In Table 2 are reported: the average thickness t of the sensors, their initial resistance $R_{0}$ before the cyclic tests, their measured resistance $R_{0s}$ before starting the monotonic test, their final resistance $R_{f}$ after the conclusion of the monotonic test and complete unloading and their *GFs*.

It should be noted that the initial resistances $R_{0}$ of the two sensors are quite similar in value. However, after completing the cyclic tests, the value of $R_{0s}$ for the sensor produced via capillary rise is increased by ~17% over $R_{0}$, whereas for the sensor produced through resin casting, it results that $R_{0s}$ is more than twice $R_{0}$. As a result, the *GF* of the former sensor is much lower than the *GF* of the latter. This behavior can be attributed to the presence of filler agglomerates exposed over the surface of the latter sensor and to the irreversible rearrangement of nanoparticles inside the agglomerates after several consecutive cycles, which produces an increase of the total resistance of the sample, as sketched in Figure 8d–f. It is also pointed out that the *GF* of the sample obtained through resin casting varies from 81 for the first monotonic test to 70 (as reported in Table 2) for the last one. Instead, in the case of samples obtained through the capillary rise method, the *GF* remains constant and equal to 27 during all five consecutive tests, demonstrating the enhanced electromechanical stability of the sensor produced through this technique and its effective potential in SHM applications.

## 4. Conclusions

Piezoresistive strain sensors made of MLG-filled polymer nanocomposite were produced through an innovative capillary rise method and fully characterized from both the morphological and electromechanical points of view.

The performances of the sensor were compared with the ones of a sensor produced through conventional mold casting and subsequent sawing of the resulting brick-shaped specimen. Both sensors were characterized by a good sensitivity at strain, with a *GF* of 27 and 70, respectively, for the sensor produced via capillary rise and for the one obtained through the resin casting method. We observed a much higher stability of the piezoresistive response of the former sensor during all flexural mechanical tests. On the contrary, a relevant deterioration of the piezoresistive properties of the latter sensor occurred after cyclic tests, performed under increasing strain levels, due to the presence of filler agglomerates exposed over the sensor surface, acting as micro-defects in the composite. The new method based on capillary rise allowed overcoming the limitations of polymer-composite strain sensors made by conventional resin casting in a mold and subsequent machining. The potentiality of the new sensor for SHM applications has been assessed.

## Figures and Tables

**Table 1:** Maximum rise height $H_{\max}$ of the plain resin and of the MLG/resin mixture, with and without hardener.

Sample	$H_{\max}$ (mm)
Plain resin	39
MLG/resin mixture	38
Plain resin with hardener	36
MLG/resin mixture with hardener	35

**Table 2:** Characteristics of the sensors produced via capillary rise and through the resin casting method: average thickness t, initial resistance $R_{0}$ before the cyclic test, starting resistance $R_{0s}$ before the monotonic test, final resistance $R_{f}$ after complete unloading and the gauge factor (*GF*).

Sensor Fabrication Method	t (µm)	$R_{0}$ (kΩ)	$R_{0s}$ (kΩ)	$R_{f}$ (kΩ)	GF
Capillary rise	157	43	50	50	27
Resin casting	163	51	103	106	70

## Abbreviations

The following abbreviations are used in this manuscript:

CNT	carbon nanotubes
SCDF	spontaneous capillary-driven filling
GF	gauge factor
GNP	graphene nanoplatelet
GO	graphene oxide
rGO	reduced graphene oxide
MLG	multilayer graphene
SEM	scanning electron microscopy
